# Do Herbaceous Species Functional Groups Have a Uniform Pattern along an Elevation Gradient? The Case of a Semi-Arid Savanna Grasslands in Southern Ethiopia

**DOI:** 10.3390/ijerph17082817

**Published:** 2020-04-19

**Authors:** Zinabu Bora, Xinwen Xu, Ayana Angassa, Yongdong Wang, Yongcheng Zhao

**Affiliations:** 1 State Key Laboratory of Desert and Oasis Ecology, Xinjiang Institute of Ecology and Geography, Chinese Academy of Sciences, Urumqi 830011, China; sms@ms.xjb.ac.cn (X.X.); zyccau@ms.xjb.ac.cn (Y.Z.); 2 University of Chinese Academy of Sciences, Beijing 100049, China; 3 Oromia Pastoral Area Development Coordination Commission, P.Box 20120 Addis Ababa, Ethiopia; 4 Department of Range and Forest Resources, Faculty of Natural Resources, Botswana University of Agriculture and Natural Resources, Private bag 0027, Gaborone, Botswana; ayana.angassa@gmail.com

**Keywords:** biomass, elevation class, functional group, herbaceous species, plant species

## Abstract

Knowledge of the total (overall) and individual herbaceous vegetation species relating to a distinctive site might help in the development of management strategies for a large number of threatened herbaceous species. This paper assesses the total and functional group herbaceous biomass, species richness, evenness, and diversity at four elevation classes in Borana rangelands of arid thorn bush savanna grasslands in Southern Ethiopia. At each elevation class, a grid of 20 × 20 m main plot was placed, and individual herbaceous species samples were collected randomly from five 1 m^2^ quadrants within the main plot. Using a single-factor analysis of variance (ANOVA), the effects of four elevation classes were considered on whole-vegetation, grasses, graminoid, and forb species diversity, evenness, richness, and biomass. A total of 49 herbaceous species were recorded. Of the total identified herbaceous species, three grass species and two graminoid species were found across all studied elevation classes, but the forb species did not overlap along the studied elevation classes. The total richness, diversity, and evenness of herbaceous species were considerable and significant along elevation classes. The grass, graminoid, and forb species richness, diversity, and evenness responded differently, and the functional group of species may be a good indicator of the community processes of grassland across elevation classes. The contribution of forb richness to the total richness was more pronounced than grass and graminoid, which indicates the shift of savanna grassland to grazing tolerant herbaceous species. The results suggest that the pooled data analysis of herbaceous vegetation community structure and biomass could obscure complicate trends of the functional group at elevation classes and for managing herbaceous species in savanna grasslands, the management models should focus on the functional group species composition, community structure, and biomass.

## 1. Introduction

Plant species are commonly grouped based on an earlier classification by means of growth form (e.g., forb, shrub), life history (e.g., evergreen, deciduous), or other morphological characteristics [1,2]. Plant life forms are widely used in community ecology and they also play an important role in ecosystem functioning, such as protection and conservation of soil and water resources, furnishing a habitat for wildlife [3]. Ecosystem functions and productivity are mainly determined by the number, nature, and relative abundance of individual species [4]. Plant community structures and productivity are usually influenced by changes in rainfall patterns [5], altitudinal gradient, increased nutrient availability, elevated atmospheric CO_2_ [6], and altered herbivory regimes [7].

Savanna grasslands are a natural ecosystem with a variety of plant species. This natural ecosystem displays stability against environmental factors and biological variables for centuries. Although the services provided by savanna grasslands are widespread, savanna grasslands are now under threat largely due to the invasion of undesirable plants [8,9]. Hassan et al. [10] stated that 22% of the world’s savanna grasslands are currently experiencing land degradation. The effects of land degradation on plant biodiversity are profound and negative since it disturbs the floristic composition, spatial distribution, and diversity [11] which results in the decline in cover and biomass production of herbaceous vegetation [12]. Others [13,14] have reported that the expansion of woody encroachment contributes indirectly to the reduction in soil organic carbon stock, total nitrogen, and changes in soil characteristics that make it more difficult for grasses to grow. Although woody plant encroachment is occurring globally across savannas, there exists significant regional variation in the magnitude of woody plant encroachment. Previous studies [14,15] have shown that the rate of woody plant encroachment in African savanna grasslands is expanding at 2.5% each decade. This is about 2.5 times that of Australian savannas [16]. According to Venter et al. [16] woody plant cover has generally increased by 7.5 million km^2^ over the last three decades in Africa with a significant reduction in the grazing capacity of rangelands [11] and loss of biodiversity at large [17]. The shift in herbaceous vegetation in savanna grasslands causes multiple ecosystem consequences, particularly in Africa with a significant reduction in grazing capacity [11] and loss of biodiversity [17], as one-fifth of the global human population depends directly on grasslands for their livelihoods [18]. Shifts in herbaceous species composition towards woody plant encroachment, the decline in the cover and biomass production of herbaceous vegetation is occurring globally across savannas [12]. The process of shifts in grassland species composition from herbaceous towards woody plant encroachment is also occurring in the savannas of Southern Ethiopia with a general decline in desirable forage species and ground cover [19,20].

Many different ecological classification systems for herbaceous vegetation have been developed, which sorts taxonomy into groups based on various alternative attributes. One of the simplest, and most common ways of herbaceous vegetation classification is the use of functional groups based on ecological similarities of species [21], i.e., functional traits of plant species [22]. Herbaceous plant species in savannas are characterized by different functional groups [23] and they have distinct life forms such as grasses, graminoid, and forb species. The distinction between graminoid and forb species has a pragmatic meaning, with grass generally being the preferred component of forage plants [24]. Most forb species are herbaceous flowering plants of various species. Moreover, forb species are less palatable with lower nutrient concentrations and higher chemical defenses as opposed to the palatable plant species such as grasses and graminoids [25]. The functional groups act as natural ecological units and that they facilitate comparisons of pollination systems at the community level. Herbaceous species composition, diversity, evenness, richness, and biomass can be seen separately or in a combination of the functional groups at different altitudes [26]. These measured characteristics of herbaceous vegetation are the most vital traits for understanding the completeness of studied ecosystems [27]. To investigate the relationship between biodiversity and ecosystem functioning, and compare the contributions of functional groups and total herbaceous species to community processes, several measures of species/community performance have been suggested. Among others, elevation gradients have become increasingly popular for investigating patterns in herbaceous vegetation community structure and biomass. It has been suggested that elevation gradients are ideal for investigating several ecological and bio-geographical hypotheses [28].

Maintenance of the savanna grasslands is mutually dependent on the presence of grazing animal and robust land use by the pastoral communities, which are often necessary to maintain the integrity of grassland ecosystems. The savanna grasslands of southern Ethiopia are a combination of arid and semi-arid environments with unpredictable patterns of rainfall and fluctuating forage productivity due to the variability of climate and change [11]. As a result, a fixed carrying capacity as predicted in a stable equilibrium model is unlikely to work in such environments [29]. Consequently, the savanna of Borana in southern Ethiopia has suffered in recent times as a result of land degradation and diminishing grassland resources putting many pastoral communities at risks of food insecurity. In traditional pastoral systems, grasslands and pastoral communities are interdependent, as grazing is often necessary to maintain the herbaceous plant community structure. Considering the importance of the Borana grasslands for the livelihood of the local communities, it is necessary to evaluate the overall and functional groups, biomass of herbaceous species, and herbaceous species community structure. Within ecology, a generalizable theory of the overall vegetation has attracted greater attention in terms of the individual functional groups in plant community structure. On the other hand, considering plant functional groups will assist in the development of appropriate management strategies for a large number of threatened herbaceous species occupying an ecosystem as a mega-diverse community. Several studies [20,30,31] have documented the status of herbaceous species composition and biomass, although those studies have rarely considered the functional groups in relation to the herbaceous species structure and biomass. There have not been any studies on the total and functional groups diversity, evenness, richness, and biomass at various elevation classes in the study areas.

Therefore, the objectives of the current study were (i) to assess the effects of elevation gradient on herbaceous species composition and functional groups distribution, (ii) to investigate the total and individual functional group diversity, evenness, richness, and biomass; (iii) to determine the relationship between the total and individual herbaceous species functional group, species richness, and biomass at different elevation classes. We hypothesized that:

**H_0_**: *Difference in elevation classes has no significant effect on the total and individual functional groups of herbaceous species composition and community structure*.

**A_1_**: *Difference in elevation classes has significant effects on the total and individual functional groups of herbaceous species diversity, evenness, richness, and biomass*.

## 2. Materials and Methods

### 2.1. Study Area

The study was conducted in the Borana rangelands of Southern Ethiopia and situated at 3°30′–5°20′ N and 37°30′–39°10′ E, about 600 km south of Addis Ababa—the capital of Ethiopia [30]. It has a slight undulating topography ranging in elevation between 700 and 1600 m above sea level (masl) with peaks up to 2000 masl [30]. The climate of the study site ranges from arid to sub-humid, with mean annual rainfall ranging between 400 and 600 mm [30]. The study area has a bimodal type of rainfall with the long rains occurring between March and May and short rains usually happen between October and November. The mean annual rainfall at the time of survey was 578.6–618.1 mm from the nearest weather stations of Yabello and Dirre, respectively (unpublished data, Ethiopian Meteorology Authority).

### 2.2. Methods

#### Preliminary Study for Site Selection

The field visit was conducted in December 2018 and four elevation classes were selected. The distribution of plant species along elevation gradients is governed by a serious of interacting biological, environmental, and historical factors. To minimize the variation among the selected elevation classes, discussions with the local herders and literature specific research on land use, land cover, soil nutrient, fire history, and land use by livestock were conducted and are summarized in Table 1 below. The Borana indigenous knowledge system recognizes the unique features of a particular landscape where they used to divide the different landscapes into the wet season, dry season, drought year, and full-year grazing landscapes [31]. The Borana pastoralists also divide the grazing lands based on the value of their animals’ contribut to the family basic needs. Accordingly, they divide their animals into the *foora-*and the *warra-*herd management for the efficient utilization of the different landscapes during different times of the year. The classification of land use by the Borana pastoralists is also acknowledged in terms of elevation classes and grazing suitability for the different species of livestock. The use of grazing lands by the *foora-*herd is usually limited to a landscape with an elevation class below 1200 masl, whereas an elevation class of above 1400 masl was grazed by the *warra-*herd. There is a transition land between the lower and upper altitude and from ecological point of view, it has an elevation range between 1200 and 1400 masl (personal communication and observation, Z. Bora). According to the Borana pastoralists, the lower elevation class was used only by livestock during the wet seasons and drought years, while the upper elevation class was used for full-year grazing and opportunistic cultivation under normal climate condition. The stocking rate for the area was rainfall dependent with boom and boost function [11] and carrying capacity used in a stable equilibrium model is generally unpredictable. The Borana elders blamed that lack of regular burning of the grasslands has promoted the proliferation of woody plant encroachment. However, a decade ago, grazers had reduced the fine fuel density and there was not sufficient herbaceous understory to support a hot fire. Even if there was herbaceous fuel as an opportunity to use fire to suppress encroaching woody plants, woody plants “escape” fires due to the ban of fireby the national government in the 1970s, and the history of fire management of the grassland in the area was hardly different.

### 2.3. Sampling Design and Data Collection

Sampling of herbaceous species was carried out from April to May 2018, which corresponds to the time of the year for peak standing biomass. By adapting the sampling scheme of Oba et al. [33] along an elevation gradient, the landscape was purposely classified into four elevation classes:

850–920 m.a.s.l (near the bottom of the valley)
1150–1200 m.a.s.l (the elevation limit for *loon foor*)
1450–1520 m.a.s.l (the elevation class of lowest situated *loon warra*), and
1690–1720 m.a.s.l (near highland).

The elevation classes from the lowest to the highest level correspond to the Roman number from I to IV, respectively. The mid-point plot location at each elevation class is indicated by county, latitude and longitude (i.e., 3°84′27.8″ N and 38°26′55.1″ E, 4°50′71.8 N and 37°74′11.3″ E, 4°57′11.4″ N and 38°27′05.3″ E, and 4°36′38.7″ N and 38°40′40.7″E from the lowest to highest elevation class, respectively). At each elevation class a grid of 20 × 20 m main plot was laid out purposely for the first grid, then, with a transect walk of 200 m intervals from the main plot with similar slope, topography and altitude five main plots were established in a consecutive way in each selected elevation class. The six main plots in each elevation classes encompassed a purposely similar slope, topography, and altitude. Within each elevation class, the main plot for accommodating herbaceous species samples in a 1 × 1 m quadrant were randomly centered at five places. The 1 × 1 m quadrants were located randomly to achieve optimal sampling of the herbaceous species. In all the four elevation classes, a total of 120 quadrants were established. In each quadrant, visual observation was used to identify the functional herbaceous plant groups (i.e., grasses, graminoids, and forbs) and the abundance of each herbaceous species was recorded using the method of [36]. All species could be identified in the field with their local names. The scientific names were obtained from taxonomic literature [37,38]. All herbaceous plants within the quadrants were clipped individually at the ground level using hand shears and each species fresh biomass was taken using sensitive beam balance. All samples were then put in a paper bag and transported to Yabello Dryland Agricultural Research Center where samples were oven-dried at 105 °C for 24 h and weighted to obtain a proxy for the above-ground primary production [39].

The following uni-variate response variables were derived from quadrant data: total herbaceous biomass (added biomass value of grass, graminoid, and forb) and each functional group biomass (grass biomass, graminoid biomass, and forb biomass). The total and functional group species diversity (Shannon–Wiener’s H’), was calculated using the Shannon–Wiener index (∑P_i_lnP_i_) _),_ whereas P_i_ = S/N, where S is the total number of species, ln is logarithm to the base e [40]. Margalef’s index was used as a simple measure of species richness [41]. Margalef’s index = (S − 1)/lnN. Where S is the total number of species, the Pielou’s Evenness Index was used [42], whereas species evenness index (e) was done by using H’/lnS.

### 2.4. Data Analyses

A single-factor analysis of variance (one-way ANOVA) (followed by Tukey Honest Significant Differences (HSD) tests when necessary) using “MASS”, “ProfileR”, “vegan”, and “coenocliner” R statistical packages (Version 3.6.0) was used to test the response variables of herbaceous (i.e., overall (total value of grasses, graminoids, and forbs)) and individual herbaceous plant functional groups (grasses, graminoids, and forbs) diversity, evenness, richness, and biomass at each studied elevation class. Statistical analyses were performed using R-software [43] and significant difference was considered at 0.05.

## 3. Results

### 3.1. Herbaceous Species Composition and Distribution

**Figure 1:**
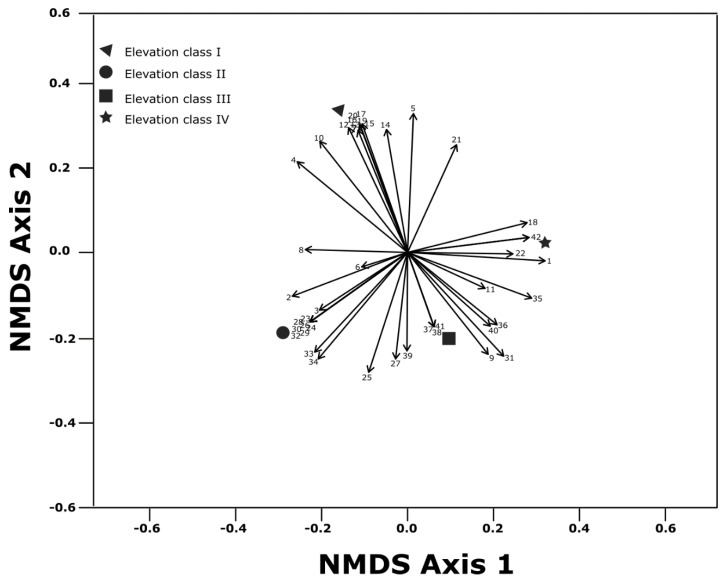
Non-metric multidimensional scaling (NMDS) ordination of floristic composition (mean species abundance per elevation class for species with a frequency > 2), associated with the four elevation classes.

A total of 49 herbaceous species were recorded across the four elevation zones (Appendix A Table A1). The results showed the highest total herbaceous species composition (55.1% or 27 different species) at elevation class II. We also recorded 24 different species of herbaceous plants (48.97%) at elevation class III, while the different herbaceous species composition at elevation classes I and IV were 23 (46.96%) and 21 (42.85%), respectively (Figure 1 and Appendix A Table A1). The distribution of herbaceous species composition across the four elevation classes was different. Of the total herbaceous species recorded, five species (10.2%) were commonly distributed across the four elevation classes; two species (4.08%) were commonly found at elevation classes I, II, and III; three species (6.12%) were recorded at elevation classes II, III, and IV; five species (10.2%) were found at elevation classes I and II; four species (8.16%) were found at elevation classes II and III; and three species (6.12%) were distributed only at elevation class III and IV while others were distributed arbitrarily across the four elevation classes. Out of the functional groups recorded, 29 different herbaceous species (59%) were grasses, nine (18.4%) were graminoid species, and 11 (22.5%) were forbs. Grass species composition were the most abundant and dominant in terms of the number of species followed by forbs. Among the 29 grasses species composition recorded, 15 grass species were commonly found at elevation classes I and III, 17 grass species were found at elevation class II, and 12 grass species at elevation class IV. Of the total identified herbaceous species, three grass species (*Eragrostis papposa*, *Pennisetum mezianum*, and *Cyperud* species) and two graminoid species, (*Chlorophytum gallabatense*, *Commelina africana*) were found at all studied elevation classes. The same forb species did not overlap across the four studied elevation classes.

### 3.2. Total and Functional Groups Herbaceous Species Community Structure

**Figure 2:**
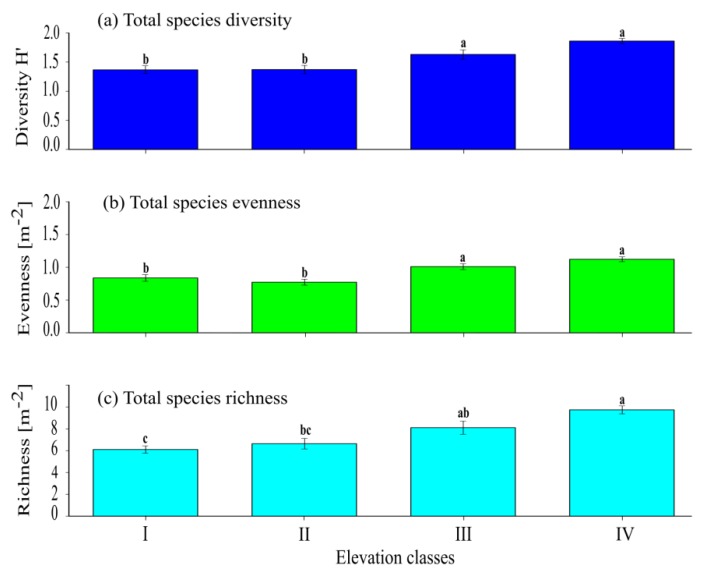
The mean (±SE) for total herbaceous species: (**a**) species diversity index; (**b**) evenness index, and (**c**) richness among the four elevation classes. The presence of significant variation among each elevation class is indicated by different superscript letters (Appendix A).

A significantly (*p <* 0.001) higher difference in terms of the total herbaceous diversity was recorded among elevation classes I, II, and IV, but the total herbaceous species diversity at elevation class IV was similar (*p* > 0.05) with the total herbaceous species diversity at elevation class III (Figure 2a and Appendix A, Table A2). The total herbaceous species diversity at elevation class III was significantly (*p* < 0.05) higher than the total herbaceous species diversity record at elevation classes I and II, while the total herbaceous species diversity between elevation classes I and II showed no significant (*p* > 0.05) difference. The total herbaceous species evenness at elevation classes I and II was lower and significantly (*p <* 0.001) higher than the total herbaceous species evenness recorded at elevation class IV. On the contrary, the total herbaceous evenness at elevation class III was significantly (*p* < 0.05) higher than the total herbaceous species evenness at elevation class I (Figure 2b). Although the total evenness increases as altitude ranges increase from elevation classes I to IV, there was no significant (*p* > 0.05) difference in terms of total herbaceous species evenness between elevation class I and II and also between elevation class III and IV. The total herbaceous species richness at elevation class IV was significantly (*p* < 0.001) higher than the total herbaceous species richness at elevation class I, and it also showed a significant (*p* < 0.05) difference with the total herbaceous species richness at elevation class II (Figure 2c). The total herbaceous species richness recorded at elevation classes III and IV did not show any significant (*p* > 0.05) difference. The total herbaceous species richness at elevation class III was significantly (*p* < 0.05) higher than the total herbaceous species richness at elevation class I, but did not show any significant (*p* > 0.05) difference in terms of total herbaceous species richness between elevation classes II and III.

### 3.3. Functional Groups and Community Structure along Elevation Classes

**Figure 3:**
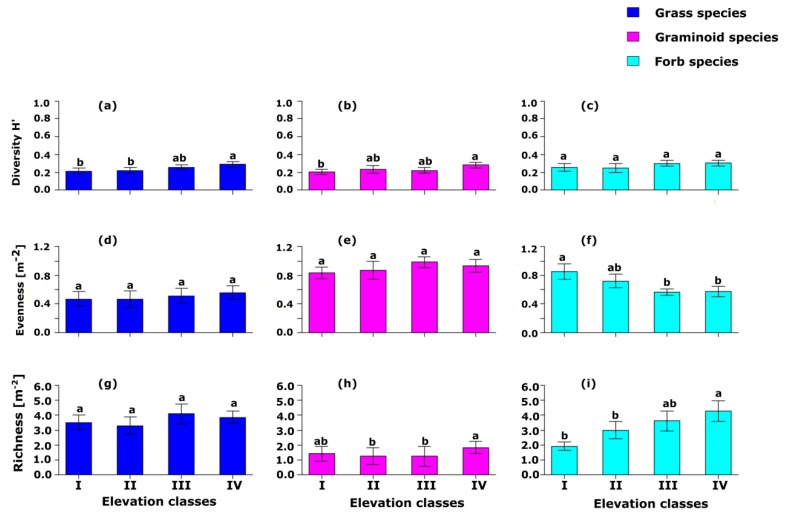
(**a**–**i**) The effects of four elevation classes on grass, graminoid, and forb herbaceous species diversity index, evenness index, and richness (m^−2^) in Borana Southern Ethiopia. Significance differences between groups based on Tukey HSD pairwise tests are indicated with different letters.

The diversity of grass species at elevation class IV was significantly (*p* < 0.05) higher than that of elevation classes I and II (Figure 3a). There was no significant (*p* > 0.05) difference in terms of grass species diversity among elevation classes I, II, and III. Similar to grass species diversity, the graminoid species diversity among elevation classes I, II, and III did not show any significant (*p* > 0.05) difference, but there is a significant (*p* < 0.05) difference between elevation classes I and IV in terms of graminoid species diversity (Figure 3b). There was a significant (*p* < 0.05) difference among the four elevation classes in terms of the diversity of forb species (Figure 3c). There was no significant (*p* > 0.05) difference among the four elevation classes in terms of the evenness of grasses and graminoid species (Figure 3d,e). However, the highest value for grass evenness was recorded at elevation class IV while the highest value of evenness for graminoid species was recorded at elevation class III. There was a significantly (*p <* 0.0001) higher forb species evenness at elevation class I than the forb species evenness at elevation classes III and IV (Figure 3f). There was no significant (*p* > 0.05) difference in grass species richness among the four studied elevation classes (Figure 3g). Generally, the highest value for grass species richness was recorded at elevation class III while the lowest value for grass species richness was recorded at elevation class II. There was a significantly (*p* < 0.0001) higher graminoid species richness at elevation class IV than the graminoid species richness recorded at elevation classes II and III (Figure 3h). There was no significant (*p >* 0.05) difference in graminoid species richness at elevation classes I, II, and III. The forb species richness at elevation classes I and II was lower and significantly (*p* < 0.001) different from forb species richness at elevation class IV (Figure 3i). However, there was no significant (*p* > 0.05) difference between elevation classes III and IV in terms of forb species richness. Similarly, there were no significant (*p* > 0.05) differences among elevation classes I, II, and III in terms of forb species richness.

### 3.4. Total and Functional Groups Biomass Productivity along Elevation Classes

The results showed a significantly (*p <* 0.001) higher total herbaceous species biomass at elevation classes II, III, and IV than elevation class I (Table 2). There was no significant (*p* > 0.05) difference among elevation classes II, III, and IV (Table 2) in terms of total herbaceous species biomass although the highest biomass was recorded at elevation class II (Table 2). The results displayed that there was a significantly (*p* < 0.05) higher grass biomass productivity at elevation class III than the grass biomass productivity recorded at elevation class I. Overall, there was no significant (*p* > 0.05) difference among elevation classes I, II, and IV in terms of grass biomass (Table 2). The results showed that the graminoid biomass productivity at elevation class II was significantly (*p* < 0.01) higher than the graminoid biomass productivity at elevation classes I and III. However, the graminoid biomass productivity at elevation class IV did not show any significant (*p* > 0.05) differences from the graminoid biomass productivity recorded at elevation classes I, III, and IV (Table 2). The forb biomass productivity was similar (*p* > 0.05) among the four elevation classes, but elevation class II had the highest biomass productivity followed by elevation classes III, I, and IV, respectively.

### 3.5. The Total and Functional Groups Richness and Biomass along Elevation

**Figure 4:**
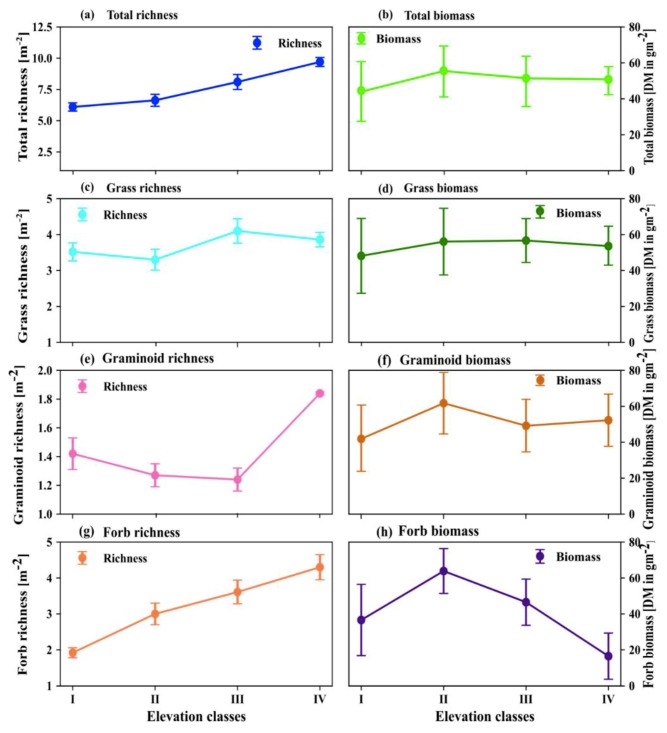
The effects of elevation classes on herbaceous species richness (**a**,**c**,**e**,**g**) and biomass of (**b**,**d**,**f**,**h**) total, grasses, graminoids, and forbs, respectively, at four elevation classes. In each elevation class the error bars tails extend from the bars to down and upper represent the lowest and highest values and the circle in the middle of the error bar indicates the median.

As indicated in Figure 4a–h, the total herbaceous species richness and total biomass productivity recorded at elevation class I was the lowest relative to the total herbaceous species richness and total biomass recorded in the other elevation classes. The highest total biomass productivity was recorded at elevation class II (Figure 4b). Generally, the total herbaceous species richness recorded at elevation class II was the third highest among the four elevation classes (Figure 4a). The highest total herbaceous species richness was recorded at elevation class IV(Figure 4a). On the other hand, the total herbaceous species biomass recorded at elevation class IV was the third highest among the four elevation classes (Figure 4b). Elevation class III produced the second highest total biomass productivity and total herbaceous species richness as compared to elevation classes I, II, and IV.

Grass biomass productivity was the lowest at elevation class I, while grass species richness was ranked third as compared to grass species richness recorded in the four studied elevation classes (Figure 4d). Grass species richness had the lowest value at elevation class II where grass biomass was ranked the second highest in terms of productivity in the same elevation classes compared to the values recorded across the four elevation classes (Figure 4c,d). The highest grass biomass productivity and species richness were recorded at elevation class III as compared to the other elevation classes. Grass biomass was ranked third at elevation class IV, while grass species richness was ranked second highest as compared to the values recorded among the studied elevation classes (Figure 4c,d).

The lowest and highest graminoid biomass productivity was recorded at elevation classes I and II, respectively (Figure 4f). However, at the two elevation classes, graminoid richness was ranked second and third highest values, respectively, as compared to the values recorded in other elevation classes (Figure 4e). The graminoid biomass at elevation class III was ranked as the third highest values but the richness of graminoid species at elevation III was the lowest as compared to the values recorded in the other elevation classes. At elevation class IV, the graminoid biomass was ranked as the second highest value, while graminoid species richness was ranked as the highest value among the values recorded in the four elevation classes (Figure 4e,f). Overall, the graminoid biomass and species richness recorded across the four elevation classes did not show any clear-cut trend.

The biomass production of forbs was ranked as the third highest value at elevation class I, while forb species richness was ranked as the lowest value at the same elevation class as compared to the values recorded in the other elevation classes (Figure 4g,h). Forbs’ biomass was ranked as the highest value at elevation class II, but forbs’ species richness was ranked as the third highest value at elevation class II when compared with the values recorded across the studied elevation classes. Both forbs’ biomass and forbs’ species richness were ranked as the second highest values when compared with the values recorded across the studied elevation classes.

## 4. Discussion

### 4.1. Herbaceous Species Composition and Total Community Structure

The results showed that the herbaceous layer was a major component of the savanna ecosystems in the study areas, while herbaceous species composition across the studied elevation classes was dissimilar. This is primarily because species composition can be a result of geographical conditions that include the predominance of typical species’ morphological and physiological adaptation to survive in a saturated environmental condition. The variation in altitude and status of biological filters can considerably influence species composition and distribution [44], although the precise effect is difficult to quantify due to other confounding factors [45,46]. It seems that the variability of soil conditions and climate along elevation classes might result in the different taxonomic and even within different herbaceous species functional groups. The results of this study clearly display that the functional groups in grass species composition, graminoid, and forb species distribution varies along the studied elevation levels. The highest number of herbaceous plant species functional groups recorded were the grass species followed by the forb species. The large number of grass and forb species might suggest that the grasslands are historically co-evolved with disturbance where most herbaceous species have developed grazing and fire tolerance traits. On the other hand, the dominance of grasses and forbs may reflect the unique adaptation of grasses where such functional group classifications are good indicators for comparisons of enrichment of vegetation and predictions in each ecological unit.

Most herbaceous species distribution was limited to certain elevation classes. The relative lack overlap in herbaceous species composition across elevation classes is confirmed by the fact that only five species, three grasses (*Eragrostis papposa*, *Pennisetum mezianum*, and *Cyperud* species), and two graminoids (*Hlorophytum gallabatens* and *Commelina africana*) were recorded across all elevation classes. There was no overlap along the studied elevation classes in terms of forb species. The probable explanation might be forbs are more sensitive to change in landscape unit than any other herbaceous species. Animals are better mediators and mechanisms of dispersal of grasses and graminoid seeds. Moreover, human impacts, like cattle raising, might facilitate more spread dispersal of palatable herbaceous species seed [47]. It can be predicted that species overlap will better survive compared to other species once exposed. As reported by previous studies [48,49] forb species are highly dynamic in their response to a small-scale environmental heterogeneity. Moreover, the forb species are less palatable with lower nutrient concentrations and livestock preferred grasses and graminoids over forb species [25], suggesting that forbs are not spread easily by cattle [47].

### 4.2. Herbaceous Community Structure along Elevation Classes

The observed total diversity, evenness, and richness of herbaceous species increase considerably from 1.37 (±0.06) to 1.86 (±0.04), 0.84 (±0.05) to 1.12 (±0.03), and from 6.1 (±0.33) to 9.7 (±0.36) as elevation classes increase from I to IV, respectively. Such diverse species across the various elevation classes most likely reflect the heterogeneity of environment along elevation classes. It was [50] pointed out that a significant increase in species richness and diversity along an elevation could be due to the effect of the nature of substrate especially with respect to soil nutrient-moisture availability along elevation gradient. In Nubra valley region Ladakh, a previous report [51] has shown that herbaceous species diversity and richness increase with increasing altitudes. Generally, there are two classes of mechanisms that account for the variation in vegetation community structure: (i) rates of speciation, extinction, and dispersal, referred to as dispersal–assembly mechanisms; and (ii) species differences, species (biotic) interactions, and environmental heterogeneity such as niche–assembly mechanisms [52].

The individual (i.e., grasses, graminoids, and forbs) herbaceous species richness was quite variable across the four studied elevation classes, which had an accompanied effect such as the hump-back, U-shaped, and linear relationship as elevation classes increase from I to IV, respectively. The overall value of grass, graminoid, and forb species richness make the total herbaceous species richness to form a linear relationship, which is similar with forb species richness across the different elevation classes. The contribution of forbs’ species richness to the total herbaceous species richness was significant and this is also consistent with the results of previous studies [48,49] in grassland and savanna ecosystems globally.

The results of the current study showed that the diversities of grass, graminoid, and forb species were increased as elevation classes increased. The evenness of grass and graminoid species also increased, while the evenness of forb species decreased as elevation class increased. Forb evenness had a distinct response with grass and graminoid species to elevation classes and the forb species being highly dynamic depending on environmental variability [53].

### 4.3. Herbaceous Biomass Productivity along Elevation Classes

The biomass productivity of total herbaceous species and functional group of grass and graminoid was significantly varied across the studied elevation classes, but the forb biomass showed no significant difference. Generally, it has been reported [54] that overgrazing resulted in a significant reduction in the abundance and biomass of the palatable herbaceous species in the rangelands. Heavy grazing is known to promote the abundance of forbs and annuals herbaceous species [55,56] by reducing the competition of highly palatable herbaceous species. This indicates that herbivore can change the species composition and the biomass of grazing intolerant species. In addition, this reflects the heterogeneity of moisture in soils and other biotic factors, which is primarily related to water–energy dynamics or productivity of palatable herbaceous species [56].

The relationship between herbaceous species richness and biomass (i.e., the rate of conversion of functional groups to the respective biomass per site) is crucial in savanna ecosystem where its major use was for livestock grazing. The studied elevation classes display different spatial patterns of herbaceous species richness and biomass production that indicate the increase in biomass may not automatically result in an increase in species richness and vice-versa. The highest grass species richness and biomass were obtained at elevation class III. This is most likely due to the fact that elevation class III was less disturbed and well managed among the studied elevation classes. At elevation class II, grass richness was the lowest, but the biomass was the second highest among other elevation classes. This could be related to the high level of grazing pressure on grass species by livestock at elevation class II, making it competitive between the highly desirable and desirable grass species. At elevation class III, the history of grazing intensity was at an intermediate level and both grass biomass and richness gained their peak values as compared to other elevation classes. The graminoid richness at elevation class III was ranked as the lowest value among the studied elevation classes. However, the biomass of graminoids at elevation class III was ranked the second lowest next to elevation class I. This might suggest that the competitive graminoid species only survived while other species disappeared as a result of heavy grazing pressure by livestock. Forbs’ species richness increases as elevation classes increase from I to IV. Forb species growth increases as elevation classes increase due to soil nutrient-moisture increment [57] and earlier results also indicate that soil moisture increases with an increase in elevation [50]. At the elevation class IV where the forb richness was the highest, its biomass was the lowest among the studied elevation classes. This implies that disturbance level might increase as elevation class increases and further facilitates more spread of forb species across an elevation gradient [58,59]. At moderate level of grazing intensities, the herbaceous functional groups such as grasses and graminoids may facilitate the accumulation of more species richness [60]. These findings indicate that the functional groups such as grass, graminoid, and forb species’ richness and biomass reconciled the herbivory response of the compensatory hypothesis that suggests that the intensity of grazing gradient has an effect on the response of herbaceous species richness and biomass.

## 5. Conclusions

All studied elevation classes had a certain number of exclusive species composition which may vary as a result of the difference in the interactions among geographical location or other factors that were not measured in this study, such as anthropogenic and environmental factors. Aside from variation in species composition, the proportion of herbaceous species functional groups (i.e., grasses, graminoids, and forbs) differed considerably at the four elevation classes. Our results showed that the total richness, evenness, and diversity of herbaceous species increased as elevation classes increased. On the contrary, the richness, diversity, and evenness of herbaceous vegetation responded differently to a particular elevation class. Thus, understanding the patterns and processes in each functional group of herbaceous species community structure and biomass is critical for ecosystem management and species conservation strategies. The study concludes that sustainable utilization of rangeland and conservation of biodiversity should be linked with the concept of landscape and pastoral land use practices. Landscape grazing suitability and the distribution of livestock in space and time might help to maintain and enhance the conservation of important herbaceous plant communities to improve the livelihoods of pastoral societies. Numerous environmental variables play a significant role in explaining the herbaceous vegetation community structure and biomass across elevation classes. Therefore, further studies that focus on multiple gradients may improve our understanding of the spatial distribution and community structure of individual herbaceous species across elevation classes of savanna ecosystems.

## Figures and Tables

**Table 1:** A summary of some important variability for the purposely selected elevation classes.

Elevation (masl)	Soil Type	Land Cover	Land Use	Reference
850–920	Vary from grey to black	Woodland, bushland, and grassland	The wet season grazing by the *foora*-herd management and drought year grazing	[31,32,33,34]
1150–1200	Drained black cracking clay	Woodland, bushland, and grassland	The wet season grazing by the *foora*-herd management and also for the dry season grazing during drought	[31,32,34,35]
1450–1520	Varies from light grey to brown sand-clay loam	Woodland, bushland, grassland, and cultivated land	Mainly used for encampments and grazed during full-year by the *warra*-herds and cultivation of land with cereals, maize, sorghum, teff, and cassava	[31,32,33,34]
1690–1720	Sandy clay to grey sand	Woodland and bushland, grassland, cultivated land	Mainly used for encampments and grazed during full-year by the *warra*-herds and cultivation of land with cereals, maize, sorghum, teff, and cassava	[30,31,32,33,34]

**Table 2:** The effects of elevation on biomass productivity g m^−2^ of the total and functional group herbaceous species along four elevation classes in Borana, Southern Ethiopia.

Response Variable	Elevation Classes				F-Value	*p*-Value
	I	II	III	IV		
Total	43.9 ± 2.27 ^b^	55.45 ± 1.92 ^a^	51.46 ± 1.93 ^a^	50.77 ± 1.07 ^a^	6.66	0.0003
Grass	48.1 ± 2.83 ^b^	56.09 ± 2.56 ^bc^	56.69± 1.63 ^ac^	53.62 ± 1.44 ^bc^	3.13	0.028
Graminoid	41.88 ± 2.52 ^b^	61.73 ± 2.29 ^a^	49.15 ± 3.53 ^b^	52.25 ± 1.94 ^ab^	5.15	0.003
Forb	53.37 ± 9.70 ^a^	57.08 ± 1.70 ^a^	54.72 ± 1.75 ^a^	50.63 ± 1.75 ^a^	0.23	0.875

^a,b,c^ Values with different superscripts are significantly different between and/or among the response variables within column.

## Appendix A

**Table A1:** List of herbaceous species encountered, arranged by functional group and relative frequency percentage at four elevation classes of Borana, Southern Ethiopia.

Herbaceous Species	Number Given on Spatial Distribution	Relative Frequency %			
		I	II	III	IV
Grasses					
*Aristida adoensis*	4	10.56	7.09	3.33	
*Blepharis ciliaris*	10	11.27	0.79		6.29
*Bothriochloa insculpta*	39			4.67	1.71
*Brachiaria species*	5	4.93		0.67	
*Cenchrus ciliaris*	36			9.33	5.71
*Chrysopogon aucheri*	31		6.30	12.67	11.43
*Cymbopogen commutatus*	19	1.41			
*Cynodon dactylon*	13	2.11			0.57
*Cyperus species*	9	7.75	4.72	12.00	10.86
*Andropogon chinensis*				0.67	
*Dactyloctenium aegyptium*	17	4.23			0.57
*Digitaria milanjiana*	25		3.94	6.00	
*Digitaria naghellensis*					0.57
*Urochloa trichopus*	26	0.70	1.57		
*Eragrostis papposa*	21	3.52	1.57	5.33	7.43
*Heteropogon contortus*				3.33	
*Eragrostis capitulifera*	33		2.36		
*Brachiaria eruciformis*	2	6.34	11.02		
*Sporobolus pellucidus*	30		5.51		
*Oropetium species*	3	6.34	8.66		3.43
*Panicum maximum*	41				0.57
*Panicum turpidum*	34		1.57	1.33	
*Pennisetum mezianum*	22	0.70	7.09	0.67	16.57
*Setaria verticillata*	12	4.93			
*Siege* species	37			1.33	
*Sporobolus pyramid*	23		6.30	14.67	
*Sporobolus* species	7	7.75	0.79	1.33	
*Themeda triandra*	27		1.57	2.00	
*Xerophyta humilis*	16	0.70	0.79		
Graminoids					
*Amarathus thunbergi*	18	3.52			0.57
*Anthospermum herbaceum*	35		0.79	0.67	1.71
*Indigofera volkensii*	20	0.70			
*Chionothrix latifolia*	42				0.57
*Chlorophytum gallabatense*	11	4.23	0.79	4.67	5.14
*Caralluma priogonium*	15	2.11	0.79		
*Commelina africana*	6	9.15	7.87	12.00	13.71
*Ocimum gratissimum*				0.67	
*Tribulus cistoides*	29		6.30		
Forbs					
*Chenopodium opulifoliu*	14	4.23			9.14
*Duosperm eremophilu*	24		7.87		
*Athroisma boranense*	8	2.11	1.57		
*Tagetes minuta*	38			0.67	
*Indigofera volkensii*	40			0.67	
*Portulaca species*		0.70			
*Sericocompsis pallida*					0.57
*Sericocompsis* species	1			0.67	2.29
*Aspilia mossambicensis*	32		0.79		
*Chlorophytum negellense*	28		0.79		
*Vigna vexillata*			0.79	0.67	0.57

**Table A2:** List of herbaceous species community structure of total and functional groups at four elevation classes of Borana, Southern Ethiopia**.**

Response Variables	Elevation Levels					
	I	II	III	IV	F-Value	*p*-Value
Diversity						
Total	1.366 ± 0.0629 ^b^	1.37 ± 0.072 ^b^	1.63 ± 0.078 ^a^	1.86 ± 0.044 ^a^	12.97	0
Grasses	0.2135 ± 0.0169 ^b^	0.218 ± 0.0175 ^b^	0.2568 ± 0.0126 ^ab^	0.2896 ± 0.0161 ^a^	4.892	0.00312
Graminoids	0.2031 ± 0.015 ^b^	0.235 ± 0.0209 ^ab^	0.221 ± 0.0171 ^ab^	0.282 ± 0.0169 ^a^	2.932	0.0387
Forbs	0.25432 ± 0.021 ^a^	0.2472 ± 0.0251 ^a^	0.3019 ± 0.017 ^a^	0.3034 ± 0.015 ^a^	2.127	0.101
Richness						
Total	6.1 ± 0.33 c	6.63 ± 0.48 bc	8.1 ± 0.60 ^ab^	9.7 ± 0.36 ^a^	12.71	0
Grasses	3.52 ± 0.25 ^a^	3.3 ± 0.29 ^a^	4.1 ± 0.34 ^a^	3.86 ± 0.20 ^a^	1.657	0.18
Graminoids	1.42 ± 0.11 ^ab^	1.27 ± 0.08 ^b^	1.24 ± 0.08 ^b^	1.84 ± 0.01 ^a^	5.812	0.00125
Forbs	1.92 ± 0.14 ^b^	3 ± 0.30 ^b^	3.61 ± 0.33 ^ab^	4.30 ± 0.35 ^a^	10.49	0
Evenness						
Total	0.84 ± 0.05 c	0.77 ± 0.04 bc	1 ± 0.04 ^ab^	1.12 ± 0.03 ^a^	12.92	0
Grasses	0.47 ± 0.05 ^a^	0.46 ± 0.06 ^a^	0.51 ± 0.05 ^a^	0.56 ± 0.05 ^a^	0.65	0.585
Graminoids	0.83 ± 0.04	0.87 ± 0.06	0.98 ± 0.03	0.93 ± 0.05	0.413	0.745
Forbs	0.85 ± 0.05 ^a^	0.71 ± 0.05 ^ab^	0.56 ± 0.02 ^b^	0.57 ± 0.03 ^b^	7.548	0.000157

^a,b,c^ Values with different superscripts are significantly different between and/or among the response variables within column.
